# Switching to a Bictegravir Single Tablet Regimen in Elderly People Living with HIV-1: Data Analysis from the BICTEL Cohort

**DOI:** 10.3390/diagnostics12010076

**Published:** 2021-12-29

**Authors:** Alessandro Lazzaro, Elio Gentilini Cacciola, Cristian Borrazzo, Giuseppe Pietro Innocenti, Eugenio Nelson Cavallari, Ivano Mezzaroma, Mario Falciano, Caterina Fimiani, Claudio Maria Mastroianni, Giancarlo Ceccarelli, Gabriella d’Ettorre

**Affiliations:** 1 Department of Public Health and Infectious Diseases, Sapienza University of Rome, Policlinico Umberto I of Rome, 00185 Rome, Italy; alessandro.lazzaro@uniroma1.it (A.L.); gentilini.1979701@studenti.uniroma1.it (E.G.C.); cristian.borrazzo@uniroma1.it (C.B.); giuseppepietro.innocenti@uniroma1.it (G.P.I.); eugenionelson.cavallari@uniroma1.it (E.N.C.); mario.falciano@uniroma1.it (M.F.); c.fimiani@policlinicoumberto1.it (C.F.); claudio.mastroianni@uniroma1.it (C.M.M.); giancarlo.ceccarelli@uniroma1.it (G.C.); 2 Department of Translational and Precision Medicine, Sapienza University of Rome, AOU Policlinico Umberto I of Rome, 00185 Rome, Italy; ivano.mezzaroma@uniroma1.it

**Keywords:** bictegravir, BIC/FTC/TAF, switch, HIV-1, HAART, antiretroviral, real-life, safety, efficacy

## Abstract

Bictegravir/emtricitabine/tenofovir alafenamide fumarate (BIC/FTC/TAF) is a recommended once-daily single tablet regimen for the treatment of people living with HIV-1 (PLWH). We aimed to assess efficacy, safety and tolerability of BIC/FTC/TAF among PLWH, with a specific focus on people older than 55 years. Thus, we recruited an observational retrospective real-life cohort including all PLWH who underwent a therapeutic switch to BIC/FTC/TAF, independently from the provenience treatment regimen. After 48 weeks of follow-up, 147 PLWH were included and 93 were older than 55 years. PLWH with HIV-RNA < 37 copies/mL increased from 140 to 146 (*p* < 0.033). Among the overall population, we observed an increase in CD4^+^ T cells count by 30.1% (*p*-value < 0.001), in CD8^+^ T cells count by 7.1% (*p*-value = 0.004) and in CD4^+^/CD8^+^ ratio by 21.5% (*p*-value < 0.001). Lipidic profile was characterized by decreasing total cholesterol/HDL ratio by 8% (*p*-value < 0.001) and LDL by 6.8% (*p*-value = 0.007). Total body weight increased by 1.8% (*p*-value = 0.014) and BMI by 4.2% (*p*-value < 0.001), even remaining within the healthy range. Hepatic and renal profile were not altered by the switch, nor were adverse events and/or discontinuations events detected. In conclusion, BIC/FTC/TAF is effective, safe and well tolerated in real life and among PLWH older than 55.

## 1. Introduction

The last few decades saw dramatic changes in the natural history of HIV-1 infection, which has been transformed by the advent of antiretroviral therapy (ART): nowadays, life expectancy of people living with HIV-1 (PLWH) has a quite similar length of general population, and an increasingly large share of HIV-1 population is aging [1,2,3]. Moreover, non-AIDS comorbidities appear earlier, and the pill burden rises sooner in comparison to healthy people. In such context, the option of a single-tablet regimen (STR) with high genetic barrier and low drug-drug interaction profile and toxicity seems to be the optimal choice for elderly PLWH [4,5].

Bictegravir/emtricitabine/tenofovir alafenamide fumarate (BIC/FTC/TAF), commercialized as Biktarvy^®^, is a relatively new antiretroviral STR for the treatment of HIV-1 infection, based on the second-generation integrase strand transfer inhibitor (INSTI) bictegravir [6,7,8]. Several pivotal clinical trials tested safety, efficacy and tolerability of BIC/FTC/TAF among experienced PLWH [9,10,11], but clinical data from real-life experience are still lacking, so much that phase 3 trials of new antiretrovirals were accused to not represent the global HIV-1 epidemic [12], especially the elderly population [13]. Thus, we aimed to assess efficacy, safety and tolerability of BIC/FTC/TAF among PLWH, with a specific focus on people older than 55 years.

## 2. Materials and Methods

### 2.1. Study Design

This is an observational retrospective real life cohort describing data from PLWH who switched their current ART to a single tablet regimen based on BIC/FTC/TAF.

The primary objective was to compare changes in HIV-RNA from baseline to week 48 after switching to BIC/FTC/TAF evaluating the percentage of PLWH with HIV-RNA < 37 copies/mL. Secondary objectives were: (a) Percentage of PLWH presenting virologic failure (HIV-RNA ≥ 37 copies/mL); (b) CD4^+^, CD8^+^ and CD4^+^/CD8^+^ ratio changes from baseline to week 48; (c) Metabolic profile (including lipids, renal, hepatic, body weight, body mass index [BMI]) changes from baseline to week 48; (d) Detection of neurological symptoms or clinical signs objectivable with the neurological examination referable to potential side effects of the drug; (e) Reasons to switch to BIC/FTC/TAF; (f) Adverse events related to BIC/FTC/TAF; (g) Risk factors related to interruption of treatment with BIC/FTC/TAF; (h) Adherence to BIC/FTC/TAF; (i) To evaluate the same primary and secondary objectives in the group of PLWH older than 55 years. 

The BICTEL cohort is an observational cohort including all PLWH who underwent a therapeutic switch to BIC/FTC/TAF, independently from the provenience ART regimen. Thus, PLWH who were receiving a protease inhibitor (PI), boosted or not, based regimen, or a non nucleosidic retrotranscriptase inhibitor (NNRTI) based regimen, as well as an INSTI, boosted or not, based regimen are enrolled. Data are retrospectively collected from patient’s medical records.

This study enrolled only PLWH included in the BICTEL cohort and with a follow-up of at least 48 weeks. The following information were extracted from the cohort database: demographics (age, gender), smoking, time from HIV-1 diagnosis (years), history of AIDS diagnosis, current hepatitis B virus (HBV) co-infection, former hepatitis C virus (HCV) infection, presence of co-morbidities (including diabetes, hypertension, cardiovascular diseases (CVD), chronic kidney disease (CKD), osteopenia and osteoporosis), 10 years atherosclerotic cardiovascular disease (ASCVD) risk score assessed by the ASCVD Risk Estimator Plus of the American College of Cardiology [14], number of non-ART co-medications, time with HIV-1 RNA < 50 copies/mL before switch (months), body weight, BMI, creatinine, estimated glomerular filtrate rate (eGFR) as measured by CKD-EPI formula [15], total HDL- and LDL-cholesterol, HIV-RNA, CD4^+^ T cells count, CD8^+^ T cells count, CD4^+^/CD8^+^ ratio, clinical assessment of eventually neurological side effect of the drug. A self-reported questionnaire regarding adherence to BIC/FTC/TAF was also administered at baseline and after 48 weeks. Adverse events (AEs) were classified as mild/moderate, severe or life threatening, according to the Division of AIDS (DAIDS) Classification. AEs were considered unrelated to BIC/FTC/TAF, possibly related or related, according to the physician’s evaluation.

### 2.2. Statistical Analysis

Baseline characteristics of enrolled patients were considered as median values and interquartile range (IQR), simple frequencies (#) and proportions (percentages, %), according to the variable type, continuous or categorical, respectively. Normality of variables distribution was assessed by the Shapiro–Wilk statistics. The change from baseline was assessed by row difference (week 48 value—baseline value) or mean relative difference ([week 48 value—baseline value]/baseline value). Longitudinal analysis was assessed by the paired Wilcoxon test for continuous variables and by the Fisher test for categorical variables. All tests were two-sided and a *p*-value of less than 0.05 was considered as statistically significant. All data were analyzed using RStudio (Version 1.3.1056 © 2009–2020 RStudio, PBC, Boston, MA, USA) and Microsoft Excel for Mac (Version 16.48).

### 2.3. Ethical Aspects 

The study was independently approved by the Committee of the Public Health and Infectious Diseases Department of “Sapienza” University of Rome and by the local Ethics Committee (No. of approval 0280/2021-6199, 31 March 2021). Written informed consent was obtained from each participant prior to the enrollment in the trial. 

## 3. Results

### 3.1. Demographic Features

At the end of the enrollment of the current study, a total of 147 PLWH were included in this 48-week follow-up; among them, 93 were over 55 years old. Details are summarized in Table 1.

#### 3.1.1. Overall Population

The median age was 57 years (49–61). In addition, 104 (70.7%) were males with a median of 16 years (10–22) from HIV-1 diagnosis. HIV-RNA at baseline was under the threshold of 37 copies/mL for more than 6 months in 140 (95.24%) of the patients, with an average time with HIV-RNA < 50 copies/mL before the switch of 37 months (37–48). The immunological profile was represented by a median CD4^+^ count of 584 (454–764) cells/μL, a median CD8^+^ count of 750 (580–1002) cells/μL and a mean CD4^+^/CD8^+^ ratio of 0.7 (0.6–0.83). Overall, 46 (31.3%) of all patients had a previous AIDS history according to CDC classification, while HBV and HCV co-infections were present in 12 (8.2%) and 16 (10.9%) participants, respectively. The 10 year cardiovascular risk assessed by the ASCVD score was detected with a median of 39% (20–50). Smokers were 98 (66.7%). At least one non-communicable clinical disease or co-morbidity was presented by 87 (59.2%) PLWH: the most prevalent was osteopenia in 35 (23.8%) participants, followed by hypertension in 31 (21.1%), osteoporosis in 25 (17%), type 2 diabetes and cardiovascular diseases in 12 (8.2%). Fifty-seven (38.8%) patients were receiving at least one non-ART co-medication, and 32 (21.8%) more than one. 

Overall, the vast majority of PLWH enrolled in the current study were receiving a prior PI-based ART (97 participants, 66%), while 23 (15.6%) were receiving an INSTI-based regimen and 29 (13.6%) an NNRTI-based regimen. Seven (4.8%) patients were receiving a combination of PI and INSTI. Out of 147 enrolled PLWH, only 5 (3.4%) were coming from an ABC-based regimen, with the majority of patients coming from a tenofovir-based regimen before the switch: 94 (63.9%) of them were receiving a TDF-based ART, while 33 (22.4%) were receiving a TAF-based regimen. Eleven (7.5%) patients were switched from a dual ART to BIC/FTC/TAF: 7 (4.8%) were receiving a dual therapy based on association of PI and INSTI, 3 (2%) was receiving dolutegravir + lamivudine and 1 (0.7%) was receiving lopinavir/ritonavir + lamivudine. Three (2%) patients were taking a monotherapy with darunavir/cobicistat. One (0.7%) patient was receiving atazanavir + ritonavir + zidovudine/lamivudine. The most frequent reason for switching to BIC/FTC/TAF recorded was to simplify a more-pills regimen to a single tablet regimen, 95 PLWH (64.6%). Toxicity accounted for 34 (23.1%) of the total switch. Minor causes recorded were: 14 pro-active switch (9.5%), 3 adherence improvement (2%), and 1 adverse event (0.7%).

#### 3.1.2. Over 55 Years Old Population

Among the 147 PLWH recruited into the study, 93 were older than 55. In addition, 69 (74.2%) were males with a median age of 60 years (57–64) and a median time from HIV-1 diagnosis of 19 years (13–25). HIV-RNA at baseline was <37 copies/mL for more than 6 months in 90 (96.8%) of the patients, with an average time with HIV-RNA < 50 copies/mL before the switch of 37 months (37–48). The immunological profile was represented by a median CD4^+^ count of 585 (462–728) cells/μL, a median CD8^+^ count of 750 (595–1000) cells/μL and a mean CD4^+^/CD8^+^ ratio of 0.7 (0.6–0.82). 34 (36.6%) of all elderly PLWH had a previous AIDS history according to CDC classification, while HBV and HCV co-infections were presented in 8 (8.6%) and 13 (14%) participants, respectively. The median 10 years cardiovascular risk, assessed by the ASCVD score, was 39% (19–50), and smokers were 59 (63.4%). Non-communicable clinical diseases were over-represented in this sub-sample, with 64 (68.8%) PLWH presenting at least one comorbidity: the most prevalent was osteopenia in 24 (25.8%) participants, followed by hypertension in 23 (24.7%), osteoporosis in 22 (23.7%), cardiovascular diseases in 11 (11.8%) and type 2 diabetes in 10 (10.8%). Higher polypharmacy was detected among PLWH > 55 years, with 43 (46.2%) of patients receiving at least one non-ART co-medication, and 28 (30.1%) more than one. Overall, the vast majority of elderly PLWH enrolled in the current study were receiving a PI-based ART (63 participants, 67.7%), while 16 (17.2%) were receiving an INSTI-based regimen and only 10 (10.8%) an NNRTI-based regimen. Two patients (2.7%) received an ABC-based regimen, while 62 (66.7%) of them a TDF-based and 19 (20.4%) a TAF-based regimen. Eight (8.6%) patients were switched from a dual ART to BIC/FTC/TAF: 4 (4.3%) participants were receiving a combination of PI and INSTI, 3 (3.2%) were receiving dolutegravir + lamivudine and 1 (1.1%) was receiving lopinavir/ritonavir + lamivudine. Two (2.1%) patients were taking monotherapy with darunavir/cobicistat. The most frequent reason reported for the switch to BIC/FTC/TAF was simplification to a single tablet regimen (in 62 PLWH, 66.7%), followed by toxicity in 21 (22.6%), pro-active switch in 6 (6.5%), adherence improvement in 3 (3.2%) and an adverse event in 1 (1.1%).

### 3.2. Safety

No AEs attributed to the use of BIC/FTC/TAF were reported in the medical records during the follow up.

### 3.3. Immunovirological Profile

**Figure 1:**
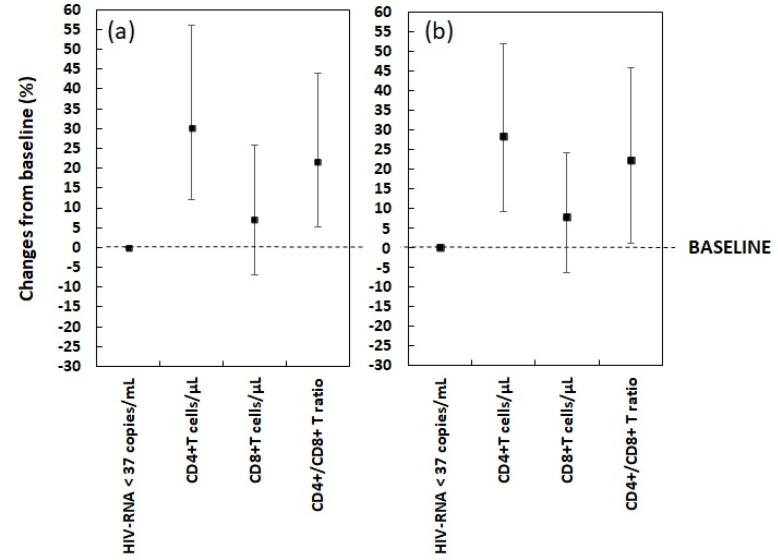
Immunovirological changes (%) from baseline: Plot report differences in immunovirological profile comparing baseline with week 48 in (**a**) overall patients and (**b**) >55 years old population. Ranked mean relative difference for each marker. Vertical bars indicate the IQR. The same axis value ranges were used in each case for ease of comparison.

After 48 weeks of follow-up, the number of participants with HIV-RNA < 37 copies/mL increased from 140 (95.3%) to 146 (99.3%), with a consequent reduction of those with HIV-RNA ≥ 37 copies/mL from 7 (4.7%) to 1 (0.7%) (*p*-value = 0.033). All 3 PLWH older than 55 with HIV-RNA ≥ 37 copies/mL at baseline showed with HIV-RNA < 37 copies/mL at week 48 (*p*-value = 0.083). A comparable improvement was detected in the immune profile. The median CD4^+^ T cells count increased from 584 (454–746) cells/μL at baseline to 767 (590–1033) cells/μL after 48 months, and CD8^+^ T cells count increased from 750 (580–1002) cells/μL to 850 (651–1032) cells/μL. Such improvements were statistically significant, with an increase in CD4^+^ T cells count by 30.1% (*p*-value < 0.001), and an increase in CD8^+^ T cells count by 7.1% (*p*-value = 0.004). A statistically significant increase (21.5%) in CD4^+^/CD8^+^ ratio was also detected, from 0.7 (0.6–0.83) to 0.9 (0.8–1) (*p*-value < 0.001). Similar results were observed among PLWH older than 55 years. Among them, CD4^+^ T cells count increased from 585 (462–728) cells/μL to 762 (589–956) cells/μL, and CD8^+^ T cells count increased from 750 (595–1000) cells/μL to 850 (677–1000) cells/μL. The improvement in CD4^+^ T cells count (increase by 28.3%) was statistically significant (*p*-value < 0.001), as well as the CD8^+^ T cells count change (increase by 3.4%) (*p*-value < 0.001). CD4^+^/CD8^+^ ratio improved significantly by 22.2% from 0.7 (0.6–0.82) to 0.9 (0.8–1) at week 48 (*p*-value < 0.001)—Table 2 and Figure 1.

### 3.4. Metabolic Profile

#### 3.4.1. Lipidic Profile, Body Weight and BMI

A significant change in the lipidic profile was detected at the end of the 48 weeks observation. In the overall population, among the fasting lipid parameters evaluated, total cholesterol levels significantly decreased by 6.9% from the median value of 190 (168–212) mg/dL to 178 (155–202) mg/dL (*p*-value < 0.001), with a significant LDL component decrease of 6.8% from 108 (88–133) mg/dL to 100 (84–131) mg/dL (*p*-value = 0.007). HDL cholesterol significantly increased by 4.4% from 49 (41–60) mg/dL to 50 (44–62) mg/dL (*p*-value = 0.033). Such changes were reflected into the total cholesterol/HDL ratio, which decreased by 8% from 3.77 (3.12–4.71) to 3.40 (2.91–4.19) (*p*-value < 0.001). Changes in total body weight were represented by a significant increase of 1.8%, rising from 77 (69–83) kg to 79 (71–85) kg (*p*-value = 0.014), with a parallel increase of 4.2% in BMI from 22 (20–24) to 22 (21–25) (*p*-value < 0.001).

Similar results were detectable among over 55 PLWH. Total cholesterol levels significantly decreased by 6.9% from 191 (169–214) mg/dL to 180 (156–203) mg/dL (*p*-value < 0.001), with a parallel significant LDL component decrease of 6.7% (*p*-value = 0.043). HDL cholesterol significantly increased by 6.8% from 46 (40–57) mg/dL to 50 (43–61) mg/dL (*p*-value = 0.027), and the total cholesterol/HDL ratio decreased by 8.6% from 3.85 (3.37–4.74) to 3.48 (2.93–4.24) (*p*-value < 0.001). Again, an increase in total body weight by 1.9% from 77 (69–86) kg to 78 (71–88) was observed, even if not statistically significant (*p* = 0.062), while BMI increased significantly by 0% (0%–5.6%) from 22 (21–24) to 22 (21–23.25) (*p*-value = 0.025)—Table 2.

#### 3.4.2. Renal Function

Interestingly, despite a significant augment of 0.8% observed in serum creatinine levels, which increased from 0.93 (0.84–1.04) mg/dL to 1.00 (0.84–1.12) mg/dL (*p*-value = 0.014), no change (0%) was detected in eGFR estimated by CKD-EPI score, which showed levels of 86 mL/min/1.73 m^2^ both at baseline (76–96) and at week 48 (72–95) (*p*-value = 0.784). 

Such trends were not detectable among over 55 PLWH, where nor serum creatinine nor eGFR showed significant changes along time (change from baseline: 0%): serum creatinine level increased from 0.9 (0.81–1.01) mg/dL to 0.99 (0.82–1.11) mg/dL (*p*-value = 0.073); eGFR estimated by CKD-EPI score decreased from 83 (74–91) to 82 (72–91) ml/min/1.73 m^2^ (*p*-value = 0.737)—Table 2.

#### 3.4.3. Hepatic Profile

Hepatic profile was safe, and no significant differences were detected in AST and ALT levels from baseline to week 48 among both overall and over 55 PLWH. Among the overall population, AST and ALT non-significantly decreased from 21 mg/dL to 20 mg/dL (change of −2.2% and −5%, respectively) from baseline (AST: 17–24; ALT: 16–26) to week 48 (AST: 16–23; ALT: 16–25) (AST *p*-value = 0.129; ALT *p*-value = 0.143). Similarly, among over 55 PLWH, AST and ALT non-significantly decreased from 21 mg/dL to 20 mg/dL (change of −5.8% and −5.9%, respectively) from baseline (AST: 18–24; ALT: 16–25) to week 48 (AST: 17–23; ALT: 16–25) (AST *p*-value = 0.149; ALT *p*-value = 0.139)—Table 2.

### 3.5. Neurological Assessment

During the period considered in the current study, we did not observe new-onset neurological disturbances such as headache, insomnia, psychiatric conditions, epilepsy.

### 3.6. Patients’ Self Reported Adherence

All 147 study participants were administered a self-reported questionnaire about adherence to current ART regimen both at baseline and at week 48. In the overall population, baseline median adherence was 95% (95–99), and a significant increase by 4.2% to 99% (95–99) was detected (*p*-value < 0.001). Similar results were observed among over 55 PLWH (Table 2).

## 4. Discussion

BIC/FTC/TAF is a recommended once-daily STR antiretroviral regimen for the treatment of both naïve and experienced PLWH. Results deriving from our real-life switching experience confirmed the efficacy and tolerability of BIC/FTC/TAF over 48 weeks. Of note, no discontinuation occurred along the entire study period and adherence level to therapy significantly increased, as assessed by patients’ self-reported questionnaire. This remarkable result is firstly related to the virological efficacy of BIC/FTC/TAF. In line with switching studies which documented non-inferiority of BIC/FTC/TAF to its comparators [9,10], percentages of PLWH with serum viral load ≥ 37 copies/mL did not increase from baseline to week 48. On top of that, we observed a significant increase of PLWH with serum viral load < 37 copies/mL among the overall population, and this trend was particularly noticeable among the over 55 PLWH, even if not reaching statistical significance (*p*-value = 0.083).

Significant changes in the immune profile were detected, with a remarkable improvement of CD4^+^ T cells count, CD8^+^ T cells count and CD4^+^/CD8^+^ ratio among both the overall population and over 55 PLWH. Our observations that confirm a positive effect of BIC/FTC/TAF among elderly PLWH is reassuring, considering that aging is known to be accompanied by immune senescence, with the CD4^+^/CD8^+^ ratio being a strong indicator of frailty and multi-morbidity [16,17].

The absence of discontinuation events recorded in our real-life experience is probably also due to the absence of AEs, not reported by receivers during the 48 weeks of observation. The study design focused on Central Nervous System (CNS)-related AEs, with a headache being the most frequent AE reported by pivotal studies and the entire INSTI class being under observation for side effects of neuro-psychiatric interest [18]. Nonetheless, during the period considered in the current study, we did not observe any new-onset of CNS-related signs or symptoms. Similarly, nausea, diarrhoea and flatulence, the most common treatment-related AEs documented in pivotal trials at week 48 (usually with an incidence lower than with the competitor drug [10,11]), were not reported in our study, thus confirming the low association of BIC/FTC/TAF to bothersome symptoms observed elsewhere [19]. Accordingly, the hepatic profile did not change from baseline to week 48.

The renal tolerability and safety of BIC/FTC/TAF documented by our longitudinal analysis is in line with data available from literature. We detected a significant increase in creatinine among the overall population, not accompanied by a parallel decrease of eGFR. Bictegravir inhibits creatinine OCT2- and MATE1-mediated tubular secretion [5,6], thus accounting for the increase in serum creatinine levels detectable after switching to BIC/FTC/TAF. Notably, such increase is more evident when switching from a PI-based regimen than from an INSTI-based regimen [9,10]. Our population was mainly composed by PI-receiving PLWH before switch (66% of the overall population and 67.7% of the over 55 sub-population), and this could have influenced the significant increase in serum creatinine levels reported in our study. Anyway, median values of eGFR were higher than the threshold of 30 mL/min, which discourages BIC/FTC/TAF use [5,6], and no relevant clinical effect on renal function was detected. Moreover, the above cited significant increase in serum creatinine level, observed in the overall population, was not documented among over 55 PLWH, whose eGFR did not change from baseline to week 48, thus confirming renal safety of BIC/FTC/TAF among elderly people.

Among fasting lipid parameters evaluated, we documented a global improvement of the lipid profile, characterized by a significant reduction of total cholesterol, LDL cholesterol and total cholesterol/HDL ratio, with a parallel increase in HDL cholesterol. Specifically, the reduction of total cholesterol as well as of total cholesterol/HDL ratio are consistent with findings at week 48 from pivotal studies comparing ART-experienced PLWH switching to BIC/FTC/TAF from boosted PI-based regimen [9,10]. Remarkably, the findings from these studies were thought to be more dependent on the influence of NRTI accompanying the PI-based regimen rather than by the switch to BIC/FTC/TAF, since switching from a tenofovir-containing regimen seemed to not affect lipid profile while switching from an abacavir/lamivudine-containing regimen significantly improved fasting lipid parameters [20]. Nonetheless, our study population included only 5 abacavir-receiving PLWH, thus suggesting that the positive effect after switch on lipid profile should not be accounted to the nucleoside backbone but to the switch to BIC/FTC/TAF itself. Interestingly, 48-week results from an ongoing study to assess the efficacy and safety of switching elderly (>65 years old) PLWH to BIC/FTC/TAF report no clinically relevant changes from baseline to week 48 in fasting lipid parameters, despite agreeing with our results on the increase in serum creatinine levels and body weight [21].

Indeed, despite the improvement in the lipid profile, after 48 weeks, our study participants experienced a significant increase in median body weight and a slight BMI increase, except among over 55 PLWH whose body weight increase did not reach statistical significance, probably due to a lack of statistical power. Several real-life experience clinical studies are also reporting warning effects of new ARV regimens on body weight and BMI and INSTIs are associated with more weight gain than PIs and NNRTIs, and dolutegravir and bictegravir more than elvitegravir/cobicistat [22]. However, despite the discussed changes from baseline, the BMI remained within the normal range. Thus, even if attention to weight gain is mandatory when selecting antiretroviral regimens, since it is associated with long-term health consequences, taken together, these data suggest the safety of the regimen containing BIC/FTC/TAF. 

## 5. Conclusions

Although further studies with larger sample size and an adequate follow-up time are needed, our 48 weeks real-life results show that the BIC/FTC/TAF regimen is an effective and tolerable choice for the treatment of virologically suppressed PLWH, regardless of age. All PLWH maintained the virological suppression with a significant improvement of the immune profile. Moreover, we detected an improvement in immune activation profile, reflected by the increase in CD4^+^/CD8^+^ ratio. Renal, hepatic and fasting lipid profile did not show any clinically significant changes during the follow up. A slight increase in body weight and BMI was detected, even if BMI remained within the healthy range. Finally, this STR helps the patients to maintain a high adherence profile as depicted by the results of the patients’ self-reported questionnaires, showing higher adherence compared to baseline.

## Figures and Tables

**Table 1:** Demographics of overall and over 55 years old population.

	**Overall** *n* = 147		Over 55 Years *n* = 93	
	m or #	IQR or %	m or #	IQR or %
**Age** (Years)	57	49–61	60	57–64
**Gender** (Male)	104	70.7%	69	74.2%
**Smoking** (Yes)	98	66.7%	59	63.4%
**Time from HIV-1 diagnosis** (Years)	16	10–22	19	13–25
**HIV-RNA < 50 cp/mL before switch** (Months)	37	37–48	37	37–48
**History of AIDS diagnosis** (Yes)	46	31.3%	34	36.6%
**HBV co-infection** (Yes)	12	8.2%	8	8.6%
**Former HCV infection** (Yes)	16	10.9%	13	14%
**HIV-1-related non-AIDS comorbidities**				
>=1	87	59.2%	64	68.8%
How many	1	0–1	1	0–2
Osteopenia	35	23.8%	24	25.8%
Ostoporosis	25	17%	22	23.7%
Type 2 Diabetes	12	8.2%	10	10.8%
Hypertension	31	21.1%	23	24.7%
Cardiovascular disease	12	8.2%	11	11.8%
**ASCVD risk score** (%)	39	20–50	39	19–50
**Other than ART co-medications**				
>=1	57	38.8%	43	46.2%
>=2	32	21.8%	28	30.%
**Pre-switch ART regimen**				
INSTI	23	15.6%	16	17.2%
NNRTI	20	13.6%	10	10.8%
PI	97	66%	63	67.7%
PI + INSTI	7	4.8%	4	4.3%
TDF-based backbone	94	63.9%	62	66.7%
TAF-based backbone	33	22.4%	19	20.4%
ABC-based backcone	5	3.4%	2	2.2%
Dual therapy	11	7.5%	8	8.6%
**Reason to switch**				
Adherence	3	2%	3	3.2%
Adverse events	1	0.7%	1	1.1%
Pro-active	14	9.5%	6	6.5%
Simplification	95	64.6%	62	66.7%
Toxicity	34	23.1%	21	22.6%

m: median; #: absolute count; IQR: interquartile range; %: percentage; cp: copies.

**Table 2:** Longitudinal analysis showing immunologic and metabolic profile changes from baseline to week 48. eGFR-CKD-EPI: glomerular filtrate rate estimated by CKD-EPI formula; IQR: Interquartile range.

**Overall**	**Baseline** ***n* = ** **147**		**Week 48** ***n* = ** **147**		**Change from Baseline** **Absolute Percentage**				***p*** **-Value**
	**Median**	**IQR**	**Median**	**IQR**	**Median**	**IQR**	**Median**	**IQR**	
**CD4^+^ T cells count** (cells/μL)	584	454–746	767	590–1033	184	73–286	30.1	12.2–56.2	**<0.001**
**CD8^+^ T cells count** (cells/μL)	750	580–1002	850	651–1032	60	−50–150	7.1	−6.8–25.7	**<0.001**
**CD4^+^/CD8^+^ T cells ratio**	0.7	0.6–0.83	0.9	0.8–1	0.15	0.04–0.3	21.5	5.3–43.9	**<0.001**
**Total Cholesterol** (mg/dL)	190	168–212	178	155–202	−14	−24–3	−6.9	−13.2–1.5	**<0.001**
**LDL **(mg/dL)	108	88–133	100	84–131	−7	−17–7	−6.8	−15.6–8	**0.007**
**HDL** (mg/dL)	49	41–60	50	44–62	2	−5–8	4.4	−8.9–20.2	**0.033**
**Total Cholesterol/HDL ratio**	3.77	3.12–4.71	3.40	2.91–4.19	−0.29	−0.92–0.19	−8	−22.7–6	**<0.001**
**AST** (mg/dL)	21	17–24	20	16–23	0	−4–2	−2.2	−18.2–14.3	0.129
**ALT** (mg/dL)	21	16–26	20	16–25	−1	−4–2	−5	−18.8–12.5	0.143
**Body weight** (Kg)	77	69–83	79	71–85	1	0–3	1.8	0–3.9	**0.014**
**Body Mass Index**	22	20–24	22	21–24	1	0–1.3	4.2	0–7	**<0.001**
**Creatinine** (mg/dL)	0.93	0.84–1.04	1	0.84–1.12	0.01	−0.01–0.08	0.8	−0.9–7.8	**0.014**
**eGFR-CKD-EPI** (mL/min/1.73 m^2^)	86	76–96	86	72–95	0	−5–5	0	−5.6–5.8	0.784
**Level of Adherence** (%)	95	95–99	99	99–99	4	0–4	4.2	0–4.2	**<0.001**
**Over 55 Years**	**Baseline** ***n* = ** **93**		**Week 48** ***n* = ** **93**		**Change from Baseline** **Absolute Percentage**				***p*** **-Value**
	**Median**	**IQR**	**Median**	**IQR**	**Median**	**IQR**	**Median**	**IQR**	
**CD4^+^ T cells count** (cells/μL)	585	462–728	762	589–956	176	52–264	28.3	9.1–51.9	**<0.001**
**CD8^+^ T cells count** (cells/μL)	750	595–1000	850	677–1000	56	−54–140	7.9	−6.3–24.3	**0.010**
**CD4^+^/CD8^+^ T cells ratio**	0.7	0.6–0.82	0.9	0.8–1	0.14	0.01–0.3	22.2	1.1–46	**<0.001**
**Total Cholesterol** (mg/dL)	191	169–214	180	156–203	−14	−24–0	−6.9	−13–0	**<0.001**
**LDL** (mg/dL)	110	88–134	103	84–134	−7	−17.5–7.5	−6.7	−15.5–7.1	**0.043**
**HDL** (mg/dL)	46	40–57	50	43–61	3	−5–8	6.8	−9.1–22.4	**0.027**
**Total Cholesterol/HDL ratio**	3.85	3.37–4.74	3.48	2.93–4.24	−0.38	−1.01–0.17	−8.6	−24.8–5.5	**<0.001**
**AST** (mg/dL)	21	18–24	20	17–23	−1	−4–2	−5.8	−18.4–14.5	0.149
**ALT** (mg/dL)	21	16–25	20	16–25	−1	−4–2	−5.9	−16–9.5	0.139
**Body weight **(Kg)	77	69–86	78	71–88	1	0–3	1.9	0–3.5	0.063
**Body Mass Index**	22	21–24	22	21–23.25	0	0–1	0	0–5.6	**0.025**
**Creatinine** (mg/dL)	0.9	0.81–1.01	0.99	0.82–1.11	0	−0.02–0.08	0	−2–8.2	0.073
**eGFR-CKD-EPI** (mL/min/1.73 m^2^)	83	74–91	82	72–91	0	−5–4	0	−6.3–4.9	0.737
**Level of Adherence **(%)	95	95−99	99	99–99	4	0–4	4.2	0–4.2	**<0.001**
